# Does hypoglycaemia affect the improvement in QoL after the transition to insulin in people with type 2 diabetes?

**DOI:** 10.1007/s40618-017-0744-5

**Published:** 2017-08-12

**Authors:** T. H. Wieringa, M. de Wit, J. W. R. Twisk, F. J. Snoek

**Affiliations:** 1 Department of Medical Psychology, VU University Medical Center (VUMC), De Boelelaan 1117, 1081 HV Amsterdam, The Netherlands; 2 Amsterdam Public Health Research Institute, Amsterdam, The Netherlands; 3 Department of Clinical Epidemiology and Biostatistics, VU University Medical Center (VUMC), De Boelelaan 1089a, 1081 HV Amsterdam, The Netherlands; 4 Department of Medical Psychology, Academic Medical Center (AMC), Meibergdreef 9, 1100 DD Amsterdam, The Netherlands

**Keywords:** Diabetes symptom burden, Emotional well-being, Hypoglycaemia, Hypoglycaemia fear, Insulin initiation, Quality of life

## Abstract

**Purpose:**

Quality of Life (QoL) of insulin-naïve people with type 2 diabetes mellitus (T2DM) improves after transition to insulin. Little is known about the role of hypoglycaemia in this context. Secondary analyses of the Study of the Psychological Impact in Real care of Initiating insulin glargine Treatment (SPIRIT) aimed to investigate the relationship between hypoglycaemia and QoL when transitioning to insulin.

**Methods:**

Insulin-naïve Dutch people with T2DM in suboptimal glycaemic control (HbA1c >53 mmol/mol; 7.0%) on maximum dose of oral glucose-lowering medications were included from 363 primary care practices (*n* = 911). Participants started insulin glargine and completed QoL-questionnaires (WHO-5 Well-being Index (WHO-5; emotional well-being), Hypoglycaemia Fear Survey-worry scale (HFS-w; hypoglycaemia fear) and Diabetes Symptom Checklist-revised (DSC-r; diabetes symptom distress) at baseline, 3 and 6 months follow-up. Linear GEE analyses were used to investigate the association between symptomatic, nocturnal, severe hypoglycaemia (number of episodes during 3 months prior to visit) and QoL over time.

**Results:**

52.5% men participated, mean age 62.2 years (SD ± 10.92), and median HbA1c 67 mmol/mol (range 61–77) (8.3%). More symptomatic hypoglycaemic episodes were associated with higher HFS-w and DSC-r scores (*P* < 0.01). Experiencing multiple nocturnal or severe episodes was related to higher symptom distress as well, when compared to no episodes. These associations did not change significantly over time.

**Conclusions:**

Hypoglycaemia is associated with lower QoL in terms of hypoglycaemia fear and diabetes symptom distress. The transition to insulin does not affect this relationship, suggesting hypoglycaemia in itself has a detrimental effect on diabetes-related QoL independent of treatment regimen.

## Background

Hypoglycaemia is a common, unpredictable and potentially dangerous side effect of insulin therapy for diabetes [1]. Hypoglycaemia can be characterized as: symptomatic hypoglycaemia (an episode of hypoglycaemia which is self-treated), nocturnal hypoglycaemia (a symptomatic episode which takes place at night) and severe hypoglycaemia (an episode of hypoglycaemia in which assistance from a third party is required).

Hypoglycaemia is inversely related to quality of life (QoL) in people with T2DM [2–4]. There is consensus that insulin therapy and insulin secretagogues (e.g. sulfonylureas, meglitinides) due to their mode of action are the main drivers of hypoglycaemia in T2DM [2]. It is estimated that 51% of people with T2DM recently commenced on insulin therapy (less than 3 years) experience at least one episode of symptomatic hypoglycaemia per year, while 7% experience at least one severe hypoglycaemia [1]. Research on the impact of hypoglycaemia on the QoL of people with T2DM suggests a greater depression burden [5]. Lifestyle and daily activities can be hampered by hypoglycaemia as a result of the symptoms negatively affecting performance, and/or as a consequence of worrying about hypoglycaemia leading to avoidant, precautionary or compensatory actions aimed to minimize the risk of hypoglycaemic episodes [6]. Fear of hypoglycaemia in people with T2DM is in itself burdensome and may translate into avoidance behaviours resulting in elevated blood glucose levels, and increased risk of long-term complications [7]. We previously demonstrated that QoL improves in people with T2DM 6 months after initiation of insulin therapy, and even after 3 months for emotional well-being [8]. However, it is unknown whether this holds for those experiencing episodes of hypoglycaemia as well.

Using data from the Study of the Psychological Impact in Real care of Initiating insulin glargine Treatment (SPIRIT) [8], a prospective observational study in routine primary care, we analysed the relationship between hypoglycaemia and QoL in people with T2DM from the moment of transition to insulin glargine onwards. Insulin glargine is a long-acting insulin analogue with a more prolonged, consistent duration of action and a lower risk of hypoglycaemia compared to NPH (humane isophane) insulin [9–11]. The SPIRIT database was chosen because patients transferred from oral treatment to insulin treatment which heightened the risk of hypoglycaemia. We hypothesize that those who experience hypoglycaemia will have a less increase in QoL after transition to insulin compared to those who do not experience hypoglycaemia.

## Methods

### Participants and procedure

We used an observational longitudinal dataset for the analyses obtained from the SPIRIT [8]. Data collection took place between January 2006 and July 2008. This study examined the change in emotional well-being, diabetes symptom distress and fear of hypoglycaemia in Dutch people with T2DM who previously used a maximum dose of oral anti-hyperglycaemic medication and were in suboptimal glycaemic control (Haemoglobin A_1c_ (HbA_1c_) >53 mmol/mol; 7.0%) [12]. People who used oral anti-hyperglycaemic agents were recruited from 363 Dutch primary care practices, spread across the Netherlands. General practitioners invited eligible people to participate. Inclusion criteria were: in clinical need of initiating long-acting insulin in accordance with the directive of the Dutch College of General Practitioners (which states that insulin therapy should be initiated if, after treatment with a maximum dose of two oral agents, optimal glycaemic control (HbA1c >53 mmol/mol; 7.0%) is not achieved), and the ability to complete questionnaires.

### Measures

Measurements were conducted at baseline (moment of transition to insulin therapy, i.e. the day clinician and person with T2DM agreed on starting insulin therapy) and 3 and 6 months after initiation of insulin glargine.

#### Quality of life

*Emotional well*-*being* was assessed with the World Health Organization (WHO)-5 Well-being Index, a well-validated instrument in people with diabetes assessing emotional well-being experienced in the two preceding weeks [13]. The WHO-5 consists of five positively stated items including positive mood, vitality and general interests. Scores were transformed into 0–100, with higher scores representing better emotional well-being. A score <50 is considered indicative of low mood.

*Fear of hypoglycaemia* was assessed using the worry subscale of the Hypoglycaemia Fear Survey (HFS-w), which assesses hypoglycaemia fear experienced in the 3 months prior to filling out [7]. HFS-w scores were transformed into a 0–100 scale, with higher scores indicating more worries about hypoglycaemia.

*Diabetes symptom distress* was measured using the revised version of the Diabetes Symptom Checklist (DSC-r) that has good psychometric properties and assesses diabetes symptom distress experienced in the month prior filling out [14]. The DSC-r consists of 34 items grouped into eight symptom subscales: hyperglycaemia, hypoglycaemia, cognitive burden, fatigue, cardiovascular burden, neuropathic pain, neuropathic sensitivity and ophthalmic function [14]. Each item asks about the presence of symptoms and, if any, to the burden of this complaint (to answer on a 5-point scale). Scores are transformed into a 0–100 score. A higher score indicates higher diabetes symptom burden.

#### Demographic and medical outcomes

Demographic and clinical data were obtained through self-report: age, sex, weight, height, diabetes duration (years), previous medication use, diabetes-related complications, comorbidity and level of education.

Hypoglycaemia was self-reported as the number of episodes during 3 months prior to visit and divided into symptomatic hypoglycaemia (defined as an episode of hypoglycaemia which is self-treated by the affected individual), nocturnal (defined as a symptomatic episode which takes place at night) and severe hypoglycaemia (defined as an episode of hypoglycaemia in which assistance from a third party is required).

Glycosylated haemoglobin (HbA1c) was obtained from medical charts.

### Statistical analyses

Generalized Estimating Equations (GEE) analyses were conducted to examine the association between hypoglycaemic episodes and QoL-outcomes over time. For every outcome, a crude and an adjusted analysis was performed. Adjustments were made for age, gender, diabetes duration, HbA1c, body mass index (BMI), level of education, and the number of complications. Additionally, the interaction between time and hypoglycaemic episodes was added to both the crude and the adjusted model. Outcome variables with a skewed distribution were log transformed. Hypoglycaemic episodes were treated as categorical variables. The categories were defined according to the median of non-zero values. Categories for symptomatic hypoglycaemia were “no hypoglycaemia”, “1–3 hypoglycaemic episodes” and “≥4 hypoglycaemic episodes”. Categories for nocturnal hypoglycaemia were “no hypoglycaemia”, “1–2 hypoglycaemic episodes” and “≥3 hypoglycaemic episodes”. Categories for severe hypoglycaemia were: “no hypoglycaemia”, “1 hypoglycaemic episode” and “2 hypoglycaemic episodes”.

Multiple imputation was used for missing data [8]. All the analyses were performed on the imputed dataset. IBM SPSS 20 was used for all analyses. Because of multiple testing, a *P* value threshold of 0.01 was used for statistical significance.

## Results

A total of 1063 people with T2DM consented to participate in the study, of which 43 were found to already use insulin and 109 were not in suboptimal control (HbA1c ≤53 mmol/mol; 7.0%) [8]. These subgroups were removed from the analyses, resulting in a sample of 911 people. In the original article of SPIRIT [8], analyses were based on the intention-to-treat principle; persons who withdrew from glargine use (*n* = 99; 11%) were thus included in the analyses. In the same study, logistic regression analyses revealed that dropout was not selective. More information about this sensitivity analysis can be found in the original article [8]. For the WHO-5, missing data were 18.0 and 43.0%. For the HFS-w, missing data were noted for 28.0% of the participants at the start up to 50.0% at 6-month follow-up. For the DSC-r, these percentages were 28.0% and 50.0%, respectively [8]. Characteristics of the study population are shown in Table 1. Changes in HbA1c, QoL-outcomes and hypoglycaemia together with *P* values are described in the original article of SPIRIT [8]. Since both HFS-w scores and DSC-r scores were distributed as skewed to the right, they were analysed as log transformed in the GEE analyses.

**Table 1:** Demographics of the study population and changes in clinical outcomes and QoL during the period of study

Demographics	Baseline	3 months	6 months
*N*	911		
Gender			
Men	479 (52.5%)		
Women	432 (47.5%)		
Age (years)	62.15 ± 10.92		
Level of education			
Low	626 (68.7%)		
Average	188 (20.7%)		
High	97 (10.7%)		
Diabetes duration (years)	6.00 (3.00–9.00)		
HbA1c (mmol/mol)^a^			
Mean ± SD	72 ± 17	61 ± 11	57 ± 11
Median (25th–75th)	67 (61–77)	60 (53–67)	56 (50–63)
HbA1c (%)^a^			
Mean ± SD	8.7 ± 1.5	7.7 ± 1.0	7.4 ± 1.0
Median (25th–75th)	8.3 (7.7–9.2)	7.6 (7.0–8.3)	7.3 (6.7–7.9)
Symptomatic hypoglycaemia^b^			
0 episodes	572 (62.8%)	524 (57.6%)	514 (56.5%)
1, 2 or 3 episodes	163 (17.9%)	211 (23.2%)	232 (25.4%)
4 or more episodes	176 (19.3%)	176 (19.3%)	165 (18.1%)
Nocturnal hypoglycaemia^b^			
0 episodes	783 (86.0%)	781 (85.8%)	745 (81.8%)
1 or 2 episodes	62 (6.8%)	85 (9.4%)	110 (12.1%)
3 or more episodes	66 (7.2%)	45 (4.9%)	56 (6.1%)
Severe hypoglycaemia^b^			
0 episodes	882 (96.8%)	872 (95.7%)	860 (94.3%)
1 episode	14 (1.5%)	28 (3.1%)	27 (3.0%)
2 episodes	15 (1.6%)	11 (1.2%)	24 (2.6%)
Previous treatment^c^			
SU-derivate	697		
Other	743		
Body mass index (BMI)	30.09 ± 5.85	30.17 ± 5.80	30.51 ± 5.76
Complications			
0	648 (71.1%)		
1 or more	263 (28.9%)		
Complications^d^			
Nephropathy	21 (2.3%)		
Neuropathy	86 (9.4%)		
Retinopathy	42 (4.6%)		
Macroalbuminuria	26 (2.9%)		
Macroangiopathy	64 (7.0%)		
Microalbuminuria	110 (12.1%)		
WHO-5^e^	56.71 (25.52)	63.33 (21.22)	65.27 (20.52)
HFS-w^f^	7.69 (1.92–21.15)	5.77 (0.00–15.38)	3.85 (0.00–15.38)
DSC-r^g^	11.71 (4.71–22.14)	8.07 (3.13–17.14)	8.09 (2.88–16.04)

For dichotomous or categorical variables the absolute numbers by subgroups and the percentage compared to the overall study population are displayed. For normally distributed variables the mean and standard deviation are shown. For skewed variables the median and the 25th and 75th percentile are shown

^a^At baseline HbA1c was skewed distributed, but normally distributed at 3 and 6 months

^b^Number of episodes during 3 months prior to visit

^c^One case may use multiple oral agents

^d^One case may have multiple complications

^e^Measured as emotional well-being experienced during 2 weeks prior to visit

^f^Measured as hypoglycaemia fear experienced during 3 months prior to visit

^g^Measured as diabetes symptom distress experienced during month prior to visit

### Symptomatic hypoglycaemia

**Fig. 1:**
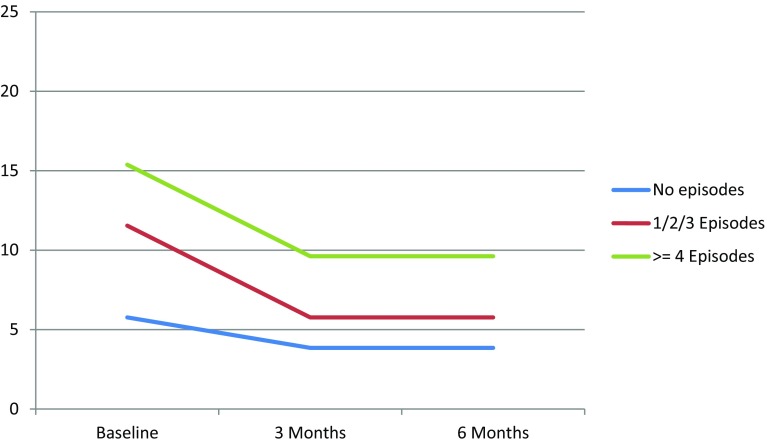
Changes in median HFS-w score per symptomatic hypoglycaemia category

**Fig. 2:**
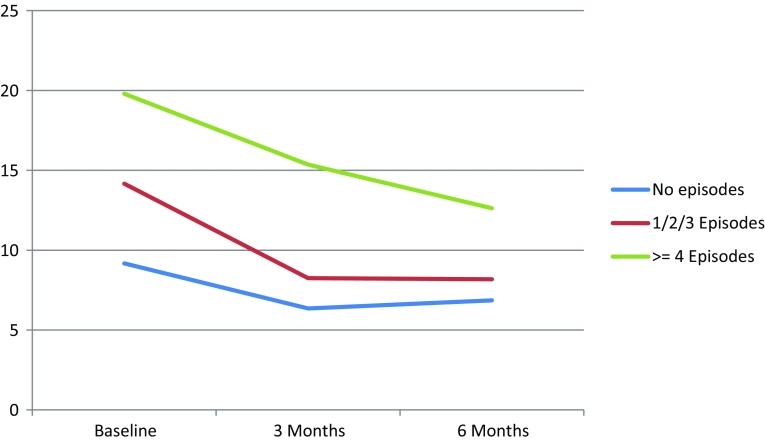
Changes in median DSC-r score per symptomatic hypoglycaemia category

Those who experienced one or more hypoglycaemic episodes reported more worries about hypoglycaemia compared to those not reporting any hypoglycaemic episode. The symptom burden of patients who experienced four or more episodes was higher compared to patients reporting fewer to no episodes (Table 2). There were no differences in emotional well-being. Changes in hypoglycaemia fear and symptom burden per symptomatic hypoglycaemia category are graphically illustrated in Figs. 1 and 2, respectively.

**Table 2:** Association between symptomatic hypoglycaemia and WHO-5, HFS-w and DSC-r

	Unadjusted model			Adjusted model^a^		
	Beta	*P* value	95%-CI	Beta	*P* value	95%-CI
WHO-5						
0–1^b^	0.33	0.565	−0.79 to 1.44	−0.27	0.673	−1.53 to 0.99
1–2^c^	−0.75	0.260	−2.05 to 0.55	−0.52	0.499	−2.02 to 0.99
0–2^d^	−0.42	0.523	−1.72 to 0.87	−0.79	0.302	−2.29 to 0.71

	Ratio of geometric averages^e^	*P* value	95%-CI	Ratio of geometric averages^e^	*P* value	95% CI
HFS-w						
0–1^b^	1.16	<0.001	1.08 to 1.24	1.24	<0.001	1.15 to 1.35
1–2^c^	1.04	0.321	0.96 to 1.13	1.07	0.174	0.97 to 1.17
0–2^d^	1.21	<0.001	1.11 to 1.31	1.33	<0.001	1.20 to 1.47
DSC-r						
0–1^b^	1.01	0.721	0.96 to 1.06	1.04	0.240	0.98 to 1.10
1–2^c^	1.08	0.004	1.03 to 1.14	1.10	0.004	1.03 to 1.17
0–2^d^	1.09	0.002	1.03 to 1.16	1.14	<0.001	1.06 to 1.22

Hypoglycaemia was self-reported as number of episodes during 3 months prior to visit. WHO-5 was self-reported as emotional well-being experienced during 2 weeks prior to visit; HFS-w as hypoglycaemia fear during 3 months prior to visit; DSC-r as diabetes symptom distress during month prior to visit

^a^Adjusted for age, diabetes duration, HbA1c, body mass index, level of education, the number of complications and gender

^b^Comparison between group 0 (no hypoglycaemia) and group 1 (1 hypoglycaemic episode) regarding severe hypoglycaemia

^c^Comparison between group 1 (1 hypoglycaemic episode) and group 2 (2 hypoglycaemic episodes) regarding severe hypoglycaemia

^d^Comparison between group 0 (no hypoglycaemia) and group 2 (2 hypoglycaemic episodes) regarding severe hypoglycaemia

^e^HFS-w and DSC-r scores were analysed as log transformed and back transformed with a ratio of geometric averages as a result. This can be interpreted as follows: “the geometric average of the reference group … times greater compared to the geometric average of the compared group”

### Nocturnal hypoglycaemia

**Fig. 3:**
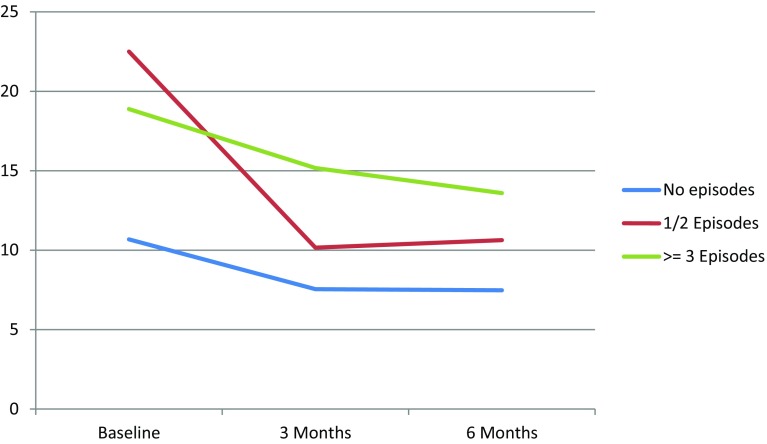
Changes in median DSC-r score per nocturnal hypoglycaemia category

DSC-r scores were significantly higher for those experiencing three or more nocturnal hypoglycaemic episodes compared to those not experiencing any nocturnal hypoglycaemia (Table 3; Fig. 3). No significant changes were found for emotional well-being and hypoglycaemia fear.

**Table 3:** Association between nocturnal hypoglycaemia and WHO-5, HFS-w and DSC-r

	Unadjusted model			Adjusted model^a^		
	Beta	*P* value	95%-CI	Beta	*P* value	95%-CI
WHO-5						
0–1^b^	1.15	0.093	−0.19 to 2.50	0.62	0.423	−0.90 to 2.14
1–2^c^	−3.03	0.002	−1.10 to −4.96	−1.97	0.059	−4.02 to 0.08
0–2^d^	−1.88	0.027	−3.53 to −0.22	−1.35	0.154	−3.20 to 0.51

	Ratio of geometric averages^e^	*P* value	95%-CI	Ratio of geometric averages^e^	*P* value	95%-CI
HFS-w						
0–1^b^	1.03	0.481	0.95 to 1.12	1.09	0.101	0.98 to 1.
1–2^c^	1.09	0.145	0.97 to 1.22	1.04	0.574	0.91 to 1.19
0–2^d^	1.12	0.015	1.02 to 1.23	1.13	0.029	1.01 to 1.26
DSC-r						
0–1^b^	1.00	0.906	0.93 to 1.07	1.05	0.255	0.97 to 1.14
1–2^c^	1.10	0.059	1.00 to 1.21	1.10	0.093	0.99 to 1.22
0–2^d^	1.09	0.029	1.01 to 1.18	1.15	0.004	1.05 to 1.26

Hypoglycaemia was self-reported as number of episodes during 3 months prior to visit. WHO-5 was self-reported as emotional well-being experienced during 2 weeks prior to visit; HFS-w as hypoglycaemia fear experienced during 3 months prior to visit; DSC-r as diabetes symptom distress experienced during month prior to visit

^a^Adjusted for age, diabetes duration, HbA1c, body mass index, level of education, the number of complications and gender

^b^Comparison between group 0 (no hypoglycaemia) and group 1 (1 or 2 hypoglycaemic episodes) regarding nocturnal hypoglycaemia

^c^Comparison between group 1 (1 or 2 hypoglycaemic episodes) and group 2 (3 or more hypoglycaemic episodes) regarding nocturnal hypoglycaemia

^d^Comparison between group 0 (no hypoglycaemia) and group 2 (3 or more hypoglycaemic episodes) regarding nocturnal hypoglycaemia

^e^HFS-w and DSC-r scores were analysed as log transformed, and back transformed with a ratio of geometric averages as a result. This can be interpreted as follows: “the geometric average of the reference group … times greater compared to the geometric average of the compared group”

### Severe hypoglycaemia

**Fig. 4:**
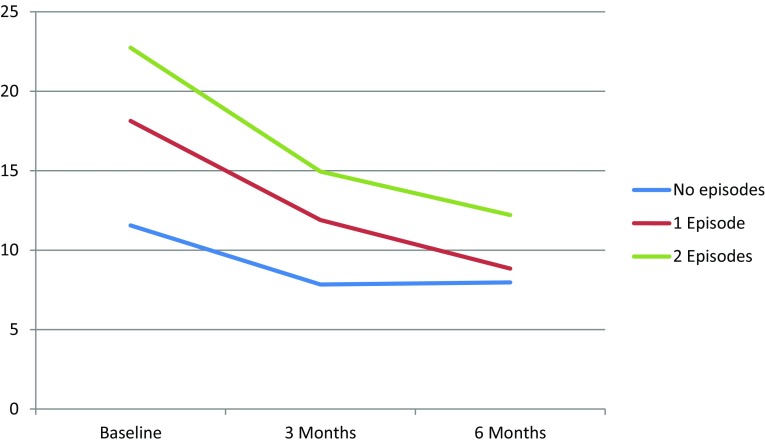
Changes in median DSC-r score per severe hypoglycaemia category

Significantly higher DSC-r scores were found for those experiencing two severe hypoglycaemic episodes compared to those not experiencing any episode (Table 4; Fig. 4). No significant changes were found for emotional well-being and hypoglycaemia fear.

**Table 4:** Association between severe hypoglycaemia and WHO-5, HFS-w and DSC-r

	Unadjusted model			Adjusted model^a^		
	Beta	*P* value	95%-CI	Beta	*P* value	95%-CI
WHO-5						
0–1^b^	1.45	0.214	−0.84 to 3.73	0.96	0.517	−1.94 to 3.86
1–2^c^	−1.77	0.286	−5.03 to 1.48	−2.58	0.224	−6.75 to 1.58
0–2^d^	−0.33	0.801	−2.87 to 2.21	−1.63	0.305	−4.73 to 1.48

	Ratio of geometric averages^e^	*P* value	95%-CI	Ratio of geometric averages^e^	*P* value	95%-CI
HFS-w						
0–1^b^	1.01	0.915	0.90 to 1.13	1.04	0.573	0.91 to 1.19
1–2^c^	1.03	0.753	0.85 to 1.26	1.09	0.480	0.86 to 1.39
0–2^d^	1.04	0.651	0.88 to 1.22	1.13	0.229	0.92 to 1.39
DSC-r						
0–1^b^	0.99	0.827	0.89 to 1.10	1.00	0.996	0.87 to 1.15
1–2^c^	1.12	0.101	0.98 to 1.27	1.16	0.068	0.99 to 1.37
0–2^d^	1.10	0.041	1.00 to 1.21	1.17	0.004	1.05 to 1.29

Hypoglycaemia was self-reported as number of episodes during 3 months prior to visit. WHO-5 was self-reported as emotional well-being experienced during 2 weeks prior to visit; HFS-w as hypoglycaemia fear experienced during 3 months prior to visit; DSC-r as diabetes symptom distress experienced during month prior to visit

^a^Adjusted for age, diabetes duration, HbA1c, body mass index, level of education, the number of complications and gender

^b^Comparison between group 0 (no hypoglycaemia) and group 1 (1,2 or 3 hypoglycaemic episodes) regarding symptomatic hypoglycaemia

^c^Comparison between group 1 (1,2 or 3 hypoglycaemic episodes) and group 2 (4 or more hypoglycaemic episodes) regarding symptomatic hypoglycaemia

^d^Comparison between group 0 (no hypoglycaemia) and group 2 (4 or more hypoglycaemic episodes) regarding symptomatic hypoglycaemia

^e^HFS-w and DSC-r scores were analysed as log transformed, and back transformed with a ratio of geometric averages as a result. This can be interpreted as follows: “the geometric average of the reference group times greater compared to the geometric average of the compared group”

In all analyses, the interaction between time and hypoglycaemic episodes was not statistically significant. This implicates that the association between hypoglycaemic events and QoL does not change over time.

## Discussion

This study aimed to assess the association between hypoglycaemia and QoL in people with T2DM on maximum dosage oral blood glucose-lowering medication and examine whether this association changes over time, after transition to insulin glargine. Hypoglycaemia was associated with lower QoL in terms of both hypoglycaemia fear and diabetes symptom distress, the diabetes-related aspects of QoL and not the overall, generic emotional well-being. The initiation of insulin therapy did not affect the relationship between hypoglycaemia and QoL. This suggests that hypoglycaemia in itself has a detrimental effect, independent of treatment regimen.

Previous studies reported severe hypoglycaemia to have a greater impact on QoL compared to symptomatic hypoglycaemia, and nocturnal hypoglycaemia to influence QoL more than daytime episodes [15]. In our study we could not demonstrate these associations, probably due to the low number of people experiencing nocturnal and severe episodes. We did find, in line with previous studies, that frequency of hypoglycaemia is negatively associated with QoL [3, 5, 15, 16]. However, further research is warranted in people with T2DM with higher incidence of severe hypoglycaemia.

The question arises whether the negative impact of hypoglycaemia on diabetes-related QoL found in this study is to be regarded clinically relevant. For diabetes symptom distress we found statistically significant increases in patients experiencing multiple hypoglycaemia episodes (both symptomatic, nocturnal and severe) and for hypoglycaemia fear we found a statistically significant increase in patients experiencing one or more symptomatic episodes compared to those with no symptomatic hypos, but very little is known about the clinical relevance of these measures. This stresses the importance of future research studying the clinical relevance of hypoglycaemia fear and changes in diabetes symptom distress.

Remarkably, hypoglycaemia rates when using oral agents are relatively high and insulin glargine is having a scarce effect on these rates. However, this is in line with previous studies [1]. The UK Hypoglycaemia Study Group (2007) found no difference in T2DM patients treated with sulfonylureas (SUs) or insulin for less than two years in both the proportion experiencing severe hypoglycaemia and the proportion experiencing mild symptomatic hypoglycaemia over a 9–12-month period [1]. A large proportion of patients in our study [76,51% (697 patients)] used SU-derivates before changing to glargine. Furthermore, Marrett et al. estimated 63% of the T2DM patients on oral medication experiencing one or more self-reported hypoglycaemic events per half a year [3]. As insulin glargine provides a more prolonged, consistent duration of action and a lower risk of hypoglycaemia compared to NPH (humane isophane) insulin [3, 4, 17], glycaemic control improves while having a scarce effect on hypoglycaemia rates.

This study has several limitations that are worth mentioning. First, hypoglycaemic episodes were self-reported. This may have resulted in underreporting of hypoglycaemia in our study population, as people with diabetes may not always recall or recognize symptoms of hypoglycaemia [18, 19] or may have limited knowledge about hypoglycaemia itself [20]. However, comparable or even lower prevalence rates of severe hypoglycaemia are found in previous research [1, 21]. Continuous glucose monitoring (CGM) could help establish the reliability of self-report, but is not feasible in a large observational study in primary care. In addition, the relatively large number of missing data is a potential weakness of observational studies, and confirmed in our study [8]. However, multiple imputation can be viewed as the most robust way of dealing with missing data [22].

This study has strengths as well. We used well-validated measures of quality of life, supporting internal validity. The study was conducted in a large and heterogeneous sample of people with T2DM in primary care settings across different regions of the country [8], which favours external validity, i.e. generalisability of our findings. We set the *p* value threshold at 0.01 to increase the probability of the findings, which can be regarded as a strength of this study as well.

## Conclusions

In conclusion, we found that hypoglycaemic episodes have a negative impact on QoL in terms of both hypoglycaemia fear and diabetes symptom distress; therefore, deserving clinical attention. We observed an impact on both hypoglycaemia fear and diabetes symptom distress with more than one episode. The initiation of insulin glargine does, however, not affect this association. Those experiencing multiple hypoglycaemic episodes report lower diabetes-related QoL compared to those not experiencing hypoglycaemia, regardless of treatment regimen. Future studies should be focused on clinically relevant changes in hypoglycaemia fear and diabetes symptom distress to interpret our findings for clinical practice. Prevention and adequate management of hypoglycaemia remains valuable and should be adequately monitored as well as the QoL of people with T2DM, with as much attention for patients using oral agents as for patients initiating insulin therapy.

## Abbreviations

DSC-r: Diabetes Symptom Checklist-revised
HbA1c: Haemoglobin A1c
HFS-w: Hypoglycaemia Fear Survey-worry subscale
QoL: Quality of life
SPIRIT: Study of the Psychological Impact in Real care of Initiating insulin glargine Treatment
T2DM: Type 2 diabetes mellitus
WHO-5: World Health Organization-5
